# Pediatric residency milestone performance is not predicted by the United States Medical Licensing Examination Step 2 Clinical Knowledge

**DOI:** 10.12688/mep.19873.1

**Published:** 2023-12-07

**Authors:** Benjamin Miller, Andrew Nowalk, Caroline Ward, Lorne Walker, Stephanie Dewar

**Affiliations:** 1 University of Pittsburgh, Pittsburgh, Pennsylvania, USA; 2 University of Oregon, Eugene, Oregon, USA

**Keywords:** USMLE, Milestones, residency performance, standardized test

## Abstract

**Background:** This study aims to show whether correlation exists between pediatric residency applicants’ quantitative scores on the United States Medical Licensing Exam Step 2 Clinical Knowledge examination and their subsequent performance in residency training based on the Accreditation Council for Graduate Medical Education Milestones, which are competency-based assessments that aim to determine residents’ ability to work unsupervised after postgraduate training. No previous literature has correlated Step 2 Clinical Knowledge scores with pediatric residency performance assessed by Milestones.

**Methods:** In this retrospective cohort study, the United States Medical Licensing Exam Step 2 Clinical Knowledge Scores and Milestones data were collected from all 188 residents enrolled in a single categorical pediatric residency program from 2012 - 2017. Pearson correlation coefficients were calculated amongst available test and milestone data points to determine correlation between test scores and clinical performance.

**Results:** Using Pearson correlation coefficients, no significant correlation was found between quantitative scores on the Step 2 Clinical Knowledge exam and average Milestones ratings (r = -0.1 for post-graduate year 1 residents and r = 0.25 for post-graduate year 3 residents).

**Conclusions:** These results demonstrate that Step 2 scores have no correlation to success in residency training as measured by progression along competency-based Milestones. This information should limit the importance residency programs place on quantitative Step 2 scores in their ranking of residency applicants. Future studies should include multiple residency programs across multiple specialties to help make these findings more generalizable.

## Introduction

The United States Medical Licensing Exam (USMLE) determines whether physicians-in-training qualify to practice medicine in the United States. The exam “assesses a physician's ability to apply knowledge, concepts, and principles, and to demonstrate fundamental patient-centered skills, that are important in health and disease and that constitute the basis of safe and effective patient care.” ^1^ The USMLE is a three-step examination, with Step 1 and Step 2 Clinical Knowledge (CK) occurring during medical school and Step 3 occurring during the first year of residency training. (From 2004 – 2019, Step 2 had a second component called Clinical Skills, which was graded on a pass-fail basis, but this has since been eliminated.) While the stated purpose of the USMLE is to determine fitness for medical licensure, it has become common practice for residency programs to use USMLE scores, especially Step 1, as an important consideration when selecting students for interview and determining rank order in the National Resident Matching Program (NRMP) Main Residency Match ^2– 5^. The NRMP uses a proprietary algorithm using applicants’ ranking of residency programs and residency programs’ ranking of applicants to provide a “match” for residency training. As the outcome of this match is contractually binding for the duration of residency training (3 years for pediatrics), this represents a very high-stakes decision.

In February 2020, the USMLE announced that it would change reporting for students’ performance on Step 1 to pass/fail ^6^. This decision was partly in response to concerns raised during the Invitational Conference on USMLE Scoring (InCUS) in 2019, regarding the use of scaled USMLE scores as predictors of, and requirements for, residency success ^7^. As part of their process, InCUS considered recent data that brought into question the role of scaled USMLE scores due to biases inherent in this standardized examination. One study showed consistent bias associated with race, age and gender in USMLE Step 1 scores, with racial bias persisting across Step 2 and 3 ^8^. The authors cited the natural implication that this bias limits opportunity in competitive specialties where Step scores may be used for screening of large numbers of applicants.

Since that time, there has been speculation about the change in relative importance of the USMLE Step 2 CK score ^9^. The USMLE has announced that Step 2 CK will remain a scaled quantitative score. The natural implication of these changes is that residency programs might simply substitute the USMLE Step 2 CK scaled score for the previously scaled score of USMLE Step 1 in their evaluation and ranking of applicants.

Logic and previous studies could substantiate this approach. Step 2 CK is allegedly more clinically relevant than Step 1 ^10, 11^, which is based more on the basic science focus of the first two years of typical American undergraduate medical education curricula. Step 2 CK is also taken during or after a student’s core clinical clerkships, which could reflect knowledge gained through clinical experiences. Some studies have shown positive correlations specifically between Step 2 CK scores and in-training exam scores ^12, 13^, certifying examinations ^14, 15^, and survey-based residency performance ^16, 17^. One study conducted in an Emergency Medicine residency found a statistically significant positive relationship between Step 2 CK scores and competency-based milestones ratings ^18^.

Conversely, some studies suggest a weaker correlation between Step 2 CK scores and Board passage than when compared to Step 1 ^19– 21^. Another study found no relevance of Step 2 CK scores when examining clinical outcomes of discrepant radiology reports ^22^.

In this study, we sought to determine if USMLE Step 2 CK scores are predictive of success in pediatric residency training. Our method of measuring “success” was the Accreditation Council for Graduate Medical Education (ACGME) Competency-Based Milestones. Milestones are reported by all accredited United States residency programs to the ACGME and are defined as “competency-based developmental outcomes (e.g., knowledge, skills, attitudes, and performance) that can be demonstrated progressively by residents/fellows from the beginning of their education through graduation to the unsupervised practice of their specialties.” ^23^ The ACGME categorizes competencies into six Domains of Competence: Patient Care, Medical Knowledge, Practice-Based Learning and Improvement, Interpersonal and Communication Skills, Professionalism, and Systems-Based Practice. The ACGME applied 21 of these competencies to pediatric training in the initial iteration of Milestones, on which this study is based: five in Patient Care, one in Medical Knowledge, four in Practice-Based Learning and Improvement, two in Interpersonal and Communication Skills, six in Professionalism, and three in Systems-Based Practice. Milestone ratings range on a scale from 1 to 5, with the exception of three competencies (Patient Care 4, Systems-Based Practice 1, and Systems-Based Practice 3) which are on a scale from 1 to 4, with increments of 0.5 between available scores.

As such, progression in Milestones is expected to mirror learners’ progression toward unsupervised practice ^24^. Studies have not only used Milestones as a measurement of individual success in residency ^25, 26^, but also as an appraisal of medical schools’ preparation of students for postgraduate training ^27^. We hypothesized that if scaled USMLE scores predict likely resident performance, then scores would correlate with ACGME Milestones ratings for residents in our program. We also compared resident performance with American Board of Pediatrics (ABP) assessments during training (In Training Examinations, or ITE) and after training (the ABP General Pediatric Certifying Examination) to determine if any of these standardized assessments have correlation with Step 2 CK or Milestones reporting.

## Methods

We reviewed and collected data from the UPMC Medical Education categorical pediatric residency program in a retrospective cohort design. This study was deemed exempt from formal review by the Institutional Review Board of the University of Pittsburgh School of Medicine because of its focus on medical education and the lack of obtaining any information from subjects that was not already available to the authors. Based at UPMC Children’s Hospital of Pittsburgh, a free standing, 313 bed tertiary care center in Pittsburgh, Pennsylvania, USA, the residency program recruits up to 30 categorical pediatric residents each year.

During the study period from 2012–2017, information was gathered on each of the 188 residents matriculated to our pediatric residency training program. We collated USMLE Step 1 and Step 2 CK scores through retrospective review of their previous residency applications. The American Board of Pediatrics annually provides ABP In-Training Exam (ITE) scores for residents in post-graduate year one (PGY1) and post-graduate year three (PGY3), as well as ABP General Pediatric Certifying Examination scores after training completion, to residency program directors; three of the authors (BM, AN, SD) accessed these data through their roles as residency program directors or associate program directors. Each of these examinations yield a scaled score between one and 300 ^1^. To assess generalizability of this study population to other pediatric residents, national data were abstracted from the National Resident Matching Program (NRMP) and ABP during the same time period ^28, 29^.

The primary endpoints of this study were the Milestone rankings at two time points during residency training: end-of-year PGY1 milestones as a measure of initial success, and end-of-year PGY3 milestones as a measure of end-of-training attainment of skills. These milestones reflect a set of key competencies central to pediatric residency training. Milestones data are generated directly from evaluations by attending physicians, pediatric fellows, supervising residents, medical students, and nurses. The only exception for this process is for two competencies (Systems-Based Practice 2 and Problem-Based Learning and Improvement 3) dealing with engagement in quality improvement; residency program directors assign these milestones ratings independently based on their assessment of specific resident performance in quality improvement endeavors. These data are then combined to create an overall average Milestone rating across all competencies, as well as an average for each domain of competence. 

We sought to measure whether performance in exam-based and milestone-based metrics correlated. Pairwise Pearson correlation coefficients were computed for every combination of USMLE Step 1, Step 2 CK, ITE and milestone ratings. For each pairwise comparison, any individual missing data for either feature was excluded for that comparison. All analyses were performed in the Python programming language with the Numpy and Pandas analysis libraries ^30^.

## Results

We examined correlation between standardized examinations before, during, and after residency training to test the hypothesis that they would be predictive of residency performance as assessed by individual pediatric Milestones data. USMLE Step 1 and Step 2 CK scores were available for 180 (97%) and 179 (96%) of residents respectively. Our program began administering the ITE for PGY-1 residents in 2015; therefore, ITE scores were available for 74 PGY-1 residents (40% of total sample, 100% of those eligible). In the final two years of our study period, two classes (PGY-1 and PGY-2) had not yet taken the PGY-3 ITE; therefore, ITE scores were available for 122 PGY-3 residents (65% of total sample, 100% of those eligible). Similarly, milestone reporting became available in 2014, naturally limiting data for some residents in the study population due to the rolling annual nature of residency matriculation. Milestones data were available for 129 PGY-1 residents (69% of total sample, 100% of those eligible) and 122 of PGY-3 residents (65% of total sample, 100% of those eligible). Our program expanded its complement of residents during the study period, thus a higher number of residents were included each year as the study progressed.

**Figure 1.**
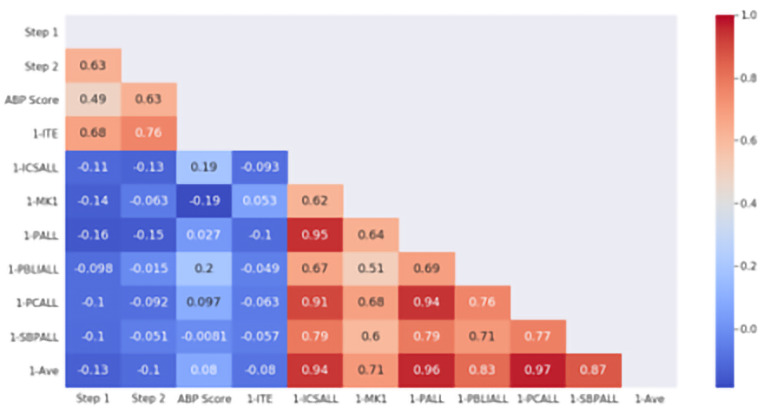
ACGME PGY-1 Milestones correlation with standardized examination. The heat map is labeled with the individual Pearson correlation coefficients between PGY1 milestones and standardized examination scores. Domains of competence (1-ICSALL = Interpersonal Communications Skills, 1-MK = Medical Knowledge, 1-PALL = Professionalism, 1-PBLIALL = Practice-Based Learning and Improvement, 1-PCALL = Patient Care, 1-SBPALL = Systems Based Practice) are shown with the overall average of all ratings (1-Ave). Standardized examinations used in correlation include USMLE Step 1 and Step 2, PGY1 In-Training Exam (1-ITE) and the American Board of Pediatrics certifying examination (ABP Score). The scale to the right demonstrates the strength of correlation (blue = negative, red = positive correlation). Note that due to timing of data collection no residents in this cohort had both 1-ITE and ABP scores available, leaving that intersecting block empty.

Figure 1 depicts the Pearson correlation coefficients between USMLE Step 2 and other standardized examinations (Step 1, ITE-1 and ABP certifying examination) and milestones averages of the PGY1 year. The heat map depicts correlation constants between averages within each domain of competence as well as overall average (1-Ave) and the examinations. No overall correlation was seen for PGY1 performance and USMLE Step 2 (r = -0.1), Step 1 (r = -0.13), or the ABP examination (r = 0.082). 

**Figure 2.**
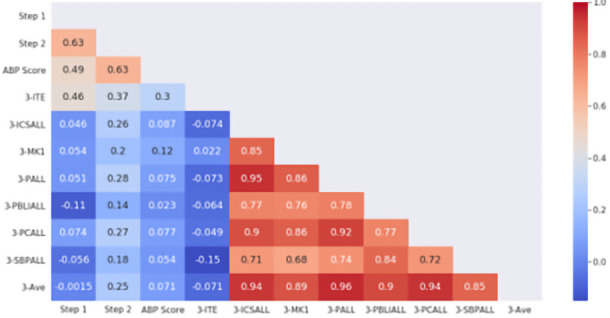
ACGME PGY-3 Milestones correlation with standardized examination. The heat map is labeled with the individual Pearson correlation coefficients between PGY3 milestones and standardized examination scores. Domains of competence (3-ICSALL = Interpersonal Communications Skills, 3-MK = Medical Knowledge, 3-PALL = Professionalism, 3-PBLIALL = Practice-Based Learning and Improvement, 3-PCALL = Patient Care, 3-SBPALL = Systems Based Practice) are shown with the overall average of all ratings (3-Ave). Standardized examinations used in correlation include USMLE Step 1 and Step 2, PGY3 In-Training Exam (3-ITE) and the American Board of Pediatrics certifying examination (ABP Score). The scale to the right demonstrates the strength of correlation (blue = negative, red = positive correlation).

When a similar comparison was made with end of PGY3 milestones assessment, as shown in Figure 2, again no correlation was observed between standardized examinations and milestones performance, either overall average or specific domains. Of specific note, overall PGY3 milestone performance did not correlate with the ITE examination at the beginning of PGY3 (r = -0.071) or ABP examination after completion of the PGY3 year (r = 0.071).

Demographics of residents included in the data set are displayed in Table 1, with comparison to ABP and NRMP data of pediatric trainees during the same period and practicing pediatrician data from the ABP. The study population was mostly female (76%), consistent with national pediatric gender distribution (71%). Residents in our program differed from average NRMP applicants during the study period in Alpha Omega Alpha medical student honor society membership (34% versus 16%) and attendance at a top 40 medical school in NIH research funding (10% versus 30%) but were similar in USMLE Step 1 and 2 scores, with an overlap in interquartile ranges.

**Table 1.** Demographics of study population compared to national data.

	CHP	All Pediatric Residents
n	188	47230 *
**Gender**		
Female	77%	71.5% *
Male	23%	28.5% *
**Race**		
African-American	5%	5.8% ^**¥**^
Asian	14%	18.2% ^**¥**^
Caucasian	75%	56.1% ^**¥**^
Other/Unknown	6%	19.1% ^**¥**^
Age	29	NA
AMG/IMG ^#^	99%/1%	75.2%/24.8% **
AOA	34%	16% **
PhD	8%	4.1% **
Other advanced degree	12%	13.6% **
Top 40 School ^	10%	29.8% **
USMLE Step 1 mean score (IQR)	236 (226-246)	227 (215-240) **
USMLE Step 2 CK mean score (SD)	250 (241-261)	243 (233-254) **
MD/DO	97%/3%	NA

* = ABP national data, 2012 – 2017 ** = 2018 NRMP data ¥ = 2020 NRMP data (first year NRMP reported information on race/ethnicity) # = American Medical School Graduate / International Medical School Graduate ^ = Top 40 US Medical School with highest NIH funding

## Discussion

This study demonstrates a lack of correlation between Step 2 CK scores and ACGME competency-based milestones performance in our categorical pediatric residency program. Additionally, we found no correlation of any standardized examination with residency performance, and examination scores only correlated with scores on other standardized examinations. These findings are consistent with previous concerns about the limitations of USMLE scores as predictors of success in residency training ^31, 32^. This suggests that residency programs should reconsider the influence of scaled USMLE Step 2 CK scores on selection of residency applicants in the National Resident Matching Program.

While there is no consensus validated measure of resident performance, competency-based milestones ratings have emerged as potentially the most reliable. Several medical disciplines, including Internal Medicine ^33^, Family Medicine ^34^, Anesthesia ^35^, and Obstetrics/Gynecology ^36^ have demonstrated reliable, consistent increase in Milestone evaluation with advancing years of training. Using milestones-based evaluations to gather data from multiple evaluators for each resident, our program has observed that overall milestone averages for residents correlate with their competence as evaluated by our Clinical Competency Committee and program leadership. As examples, high scores have correlated with chief resident selection, and low scores have correlated with remediation or termination (data not shown). Park et al had similar findings that low milestones ratings correlate with trainees who are struggling in their learning ^33^.

We also found that, while resident performance as judged by milestones ratings did not correlate with standardized examination scores, USMLE Step 2 CK scores correlated with ITE scores in the PGY1 year and with eventual ABP General Pediatric Certifying Examination scores ( Figure 1 and Figure 2). These findings are consistent with previously published studies in pediatrics ^37^ and other medical specialties ^38, 39^. This suggests that previous performance on standardized examinations correlates with performance on subsequent standardized examinations, rather than with competency-based achievement. While previous authors suggest this fact alone substantiates the use of USMLE scaled scores in the selection of residency program applicants ^38^, our data may suggest otherwise for pediatric programs, under the established assumption that educational achievement within the ACGME six domains of competence is a better measure of physician competence than whether someone is able to pass a standardized examination.

Previous authors have posited a similar belief. In separate publications, Prober *et al.* ^40^ and Bernstein ^2^ argue that not only is the USMLE being misused, but also that the misuse might be harmful. It is estimated that medical school students historically would devote weeks to studying for the USMLE Step 1 ^41^. It is accepted that residency programs across specialties use examination scores not solely for the declared purpose of competence for practice but also as a measure of acceptability into residency programs, including “filtering out” applicants below a minimum score in some circumstances. Our study suggests that while preparations for the USMLE Step 1 may have resulted in a higher score, they do not correlate with improved performance in residency training, and in fact may function at every level to limit opportunity based on race, gender, and age. In fact, correlations between Step 1 performance and milestones achievement in our program skewed slightly negative, an interesting finding.

With the shift of USMLE Step 1 from a scaled examination to pass/fail, residency program directors will likely look to other sources to differentiate between applicants. A rational replacement could be USMLE Step 2 CK. However, our data demonstrate that Step 2 CK scores are not a good predictor of performance in residency. Even though the correlation between Step 2 CK and milestones ratings are slightly stronger than between Step 1 and milestones ratings, correlation here is essentially random and uninformative for selection of potential trainees.

These data suggest that pediatric residency program directors should exercise caution in the use of any scaled USMLE score in their evaluation of program applicants. The change to scoring USMLE Step 1 on a pass/fail basis will likely incite significant cultural shifts in undergraduate medical education, including an even higher importance placed on USMLE 2 and grades in core clinical clerkships. Residency programs may need to follow suit, using these grades and other information in the Medical Student Performance Evaluation letter to determine whether to offer an interview to their program and subsequent rank for the National Resident Matching Program. While this process of filtering applicants through holistic review may be more arduous than screening based on a scaled test score, it will likely result in a more accurate determination of an applicant’s ability to achieve success in residency.

One important caveat to note is that the ACGME uses Board Certifying Examination pass rates for a pediatric training program’s graduates in their accreditation process of residency and fellowship programs ^42^. Thus, as our data suggest that scaled USMLE scores correlate with eventual board pass rate, programs are incentivized to match applicants who score well on standardized exams, which our data also demonstrate has no correlation with actual performance as a physician.

Our study has limitations. First, this is a single center, pediatric study over a short time. We only compared USMLE scores and milestones ratings for residents who matched into our training program. We do not have access to data about applicants who matched elsewhere, or residents in other training programs, pediatrics or otherwise. This limitation is somewhat mitigated by the fact that our residents are largely representative of pediatric applicants from allopathic medical schools in the United States, as noted in Table 1. Based on these comparisons to national data, the results of this study may be generalizable, but further study in other pediatric residencies and other specialties is warranted.

Second, there is wide variability in how residency programs establish Milestones ratings for their residents. We have described our method above, which incorporates input from a variety of sources to try to minimize confounding factors and mitigate potential bias, but the collection process has not been standardized among programs. Therefore, programs that generate and/or collate Milestones data differently may see disparate results from ours.

## Conclusion

This study is the first to correlate ACGME Milestones performance with standardized examination scores in pediatric residents. We show that while USMLE Step examinations correlate with future scores on ITE and Board Certifying examinations, they do not correlate with ACGME Milestones performance by residents in our program. This suggests that pediatric residency training programs should use caution in applying USMLE scores as a predictor of success in residency training. Further investigation verifying these findings in other training programs and examining applicant characteristics that may have a greater correlation with resident performance is warranted.

## Data Availability

It is not ethically feasible to share our raw data publicly. Though the names of residents included in the study have been redacted, there may be enough personal information within the dataset that could identify residents who have trained in our program. These data could include sensitive information such as scores on board-certifying examinations, and this information should not be part of the public domain. Reviewers wishing to see raw data can contact the corresponding author (Benjamin Miller, benjamin.miller@chp.edu) to request access.

## References

[1] United States Medical Licensing Examination. Accessed January 5, 2023. Reference Source

[2] Bernstein J : Not the Last Word: Ending the Residency Application Arms Race-Starting with the USMLE. *Clin Orthop Relat Res.* 2016;474(12):2571–6. 10.1007/s11999-016-5108-5 27730419 PMC5085960

[3] Green M Jones P Thomas JX Jr : Selection criteria for residency: results of a national program directors survey. *Acad Med.* 2009;84(3):362–7. 10.1097/ACM.0b013e3181970c6b 19240447

[4] London DA Kwon R Atluru A : More on How USMLE Step 1 Scores Are Challenging Academic Medicine. *Acad Med.* 2016;91(5):609–10. 10.1097/ACM.0000000000001159 27115655

[5] Schrock JB Kraeutler MJ Dayton MR : A cross-sectional analysis of minimum USMLE Step 1 and 2 criteria used by orthopaedic surgery residency programs in screening residency applications. *J Am Acad Orthop Surg.* 2017;25(6):464–468. 10.5435/JAAOS-D-16-00725 28459711

[6] George J : No More Numerical Scores for USMLE Step 1 Exam. MedPage Today, February 14,2020; accessed January 5, 2023. Reference Source

[7] Summary Report and Preliminary Recommendations from the Invitational Conference on USMLE Scoring (InCUS). March 11-12,2019; Accessed February 6, 2022. Reference Source

[8] Rubright JD Jodoin M Barone MA : Examining Demographics, Prior Academic Performance, and United States Medical Licensing Examination Scores. *Acad Med.* 2019;94(3):364–70. 10.1097/ACM.0000000000002366 30024473

[9] Nolen L Goshua A Farber ON : Cheers and jeers as med school's Step 1 test becomes pass/fail. STATNews, February 14,2020; accessed January 5, 2023. Reference Source

[10] Lee M Vermillion M : Comparative values of medical school assessments in the prediction of internship performance. *Med Teach.* 2018;40(12):1287–1292. 10.1080/0142159X.2018.1430353 29390938

[11] Cuddy MM Dillon GF Clauser BE : Assessing the Validity of the USMLE Step 2 Clinical Knowledge Examination through an Evaluation of its Clinical Relevance. *Acad Med.* 2004;79(10 Suppl):S43–S45. 10.1097/00001888-200410001-00013 15383386

[12] Perez JA Jr Greer S : Correlation of United States Medical Licensing Examination and internal medicine in-training examination performance. *Adv Health Sci Educ Theory Pract.* 2009;14(5):753–758. 10.1007/s10459-009-9158-2 19283500

[13] Spurlock DR Jr Holden C Hartranft T : Using United States Medical Licensing Examination ^®^ (USMLE) examination results to predict later in-training examination performance among general surgery residents. *J Surg Educ.* 2010;67(6):452–56. 10.1016/j.jsurg.2010.06.010 21156308

[14] Maker VK Zahedi MM Villines D : Can we predict which residents are going to pass/fail the oral boards? *J Surg Educ.* 2012;69(6):705–13. 10.1016/j.jsurg.2012.08.009 23111034

[15] Welch TR Olson BG Nelsen E : United States Medical Licensing Examination and American Board of Pediatrics Certification Examination results: Does the residency program contribute to trainee achievement. *J Pediatr.* 2017;188:270–274. e3. 10.1016/j.jpeds.2017.05.057 28629684

[16] Andriole DA Jeffe DB Whelan A : What predicts surgical internship performance? *Am J Surg.* 2004;188(2):161–64. 10.1016/j.amjsurg.2004.03.003 15249242

[17] Hamstra SJ Cuddy MM Jurich D : Exploring the Association Between USMLE Scores and ACGME Milestone Ratings: A Validity Study Using National Data From Emergency Medicine. *Acad Med.* 2021;96(9):1324–1331. 10.1097/ACM.0000000000004207 34133345 PMC8378430

[18] Sharma A Schauer DP Kelleher M : USMLE Step 2 CK: Best Predictor of Multimodal Performance in an Internal Medicine Residency. *J Grad Med Educ.* 2019;11(4):412–419. 10.4300/JGME-D-19-00099.1 31440335 PMC6699543

[19] Dillon GF Swanson DB McClintock JC : The relationship between the American Board of Anesthesiology Part 1 Certification Examination and the United States Medical Licensing Examination. *J Grad Med Educ.* 2013;5(2):276–283. 10.4300/JGME-D-12-00205.1 24404273 PMC3693694

[20] Dyrstad BW Pope D Milbrandt JC : Predictive measures of a resident’s performance on written orthopaedic board scores. *Iowa Orthop J.* 2011;31:238–43. 22096449 PMC3215143

[21] Puscas L Chang CWD Lee HJ : USMLE and otolaryngology: Predicting board performance. *Otolaryngol Head Neck Surg.* 2017;156(6):1130–35. 10.1177/0194599817702874 28418270

[22] Agarwal V Bump GM Heller MT : Do residency selection factors predict radiology resident performance? *Acad Radiol.* 2018;25(3):397–402. 10.1016/j.acra.2017.09.020 29239834

[23] Accreditation Council for Graduate Medical Education: Milestones FAQ. Accessed January 5, 2023. Reference Source

[24] Li ST : The Promise of Milestones: Are They Living Up to Our Expectations? *J Grad Med Educ.* 2017;9(1):54–7. 10.4300/JGME-D-16-00694.1 28261394 PMC5319628

[25] Marcus-Blank B Dahlke JA Braman JP : Predicting Performance of First-Year Residents: Correlations Between Structured Interview, Licensure Exam, and Competency Scores in a Multi-Institutional Study. *Acad Med.* 2019;94(3):378–387. 10.1097/ACM.0000000000002429 30157088

[26] Sozener CB Lypson ML House JB : Reporting Achievement of Medical Student Milestones to Residency Program Directors: An Educational Handover. *Acad Med.* 2016;91(5):676–84. 10.1097/ACM.0000000000000953 26488570

[27] Wancata LM Morgan H Sandhu G : Using the ACMGE Milestones as a Handover Tool from Medical School to Surgery Residency. *J Surg Educ.* 2017;74(3):519–29. 10.1016/j.jsurg.2016.10.016 27908638

[28] National Resident Matching Program. Accessed January 3, 2023. Reference Source

[29] The American Board of Pediatrics: Data and Workforce. Accessed January 3, 2023. Reference Source

[30] McKinney W : Data structures for statistical computing in Python. McKinney. *Proceedings of the 9th Python in Science Conference*.2010;445. Reference Source

[31] Gelinne A Zuckerman S Benzil D : United States Medical Licensing Exam Step I Score as a predictor of neurosurgical career beyond residency. *Neurosurgery.* 2019;84(5):1028–1037. 10.1093/neuros/nyy313 30010944

[32] Burkhardt J Parekh K Gallhue F : A critical disconnect: Residency selection factors lack correlation with intern performance. *J Grad Med Educ.* 2020;12(6):696–704. 10.4300/JGME-D-20-00013.1 33391593 PMC7771600

[33] Park YS Zar FA Norcini JJ : Competency Evaluations in the Next Accreditation System: Contributing to Guidelines and Implications. *Teach Learn Med.* 2016;28(2):135–45. 10.1080/10401334.2016.1146607 26849397

[34] Peabody MR O'Neill TR Peterson LE : Examining the Functioning and Reliability of the Family Medicine Milestones. *J Grad Med Educ.* 2017;9(1):46–53. 10.4300/JGME-D-16-00172.1 28261393 PMC5319627

[35] Ross FJ Metro DG Beaman ST : A first look at the Accreditation Council for Graduate Medical Education anesthesiology milestones: implementation of self-evaluation in a large residency program. *J Clin Anesth.* 2016;32:17–24. 10.1016/j.jclinane.2015.12.026 27290937

[36] Goldman RH Tuomala RE Bengtson JM : How Effective are New Milestones Assessments at Demonstrating Resident Growth? 1 Year of Data. *J Surg Educ.* 2017;74(1):68–73. 10.1016/j.jsurg.2016.06.009 27395399

[37] McCaskill QE Kirk JJ Barata DM : USMLE step 1 scores as a significant predictor of future board passage in pediatrics. *Ambul Pediatr.* 2007;7(2):192–5. 10.1016/j.ambp.2007.01.002 17368416

[38] Harmouche E Goyal N Pinawin A : USMLE scores predict success in ABEM initial certification: A multicenter study. *West J Emerg Med.* 2017;18(3):544–49. 10.5811/westjem.2016.12.32478 28435509 PMC5391908

[39] Swanson DB Sawhill A Holtzman KZ : Relationship between performance on part I of the American Board of Orthopaedic Surgery Certifying Examination and Scores on USMLE Steps 1 and 2. *Acad Med.* 2009;84(10 Suppl):S21–4. 10.1097/ACM.0b013e3181b37fd2 19907379

[40] Prober CG Kolars JC First LR : A Plea to Reassess the Role of United States Medical Licensing Examination Step 1 Scores in Residency Selection. *Acad Med.* 2016;91(1):12–5. 10.1097/ACM.0000000000000855 26244259

[41] Kumar AD Shah MK Maley JH : Preparing to take the USMLE Step 1: A survey on medical students’ self-reported study habits. *Postgrad Med J.* 2015;91(1075):257–261. 10.1136/postgradmedj-2014-133081 25910497

[42] ACGME Program Requirements for Pediatrics. 2021; Accessed January 5, 2023. Reference Source

