# Supplementary material for: Pediatric residency milestone performance is not predicted by the United States Medical Licensing Examination Step 2 Clinical Knowledge
Source: MedEdPublish (2016). 2024 Aug 22;13:308. Originally published 2023 Dec 7. [Version 2] doi: 10.12688/mep.19873.2 (PMC11344197; doi:10.12688/mep.19873.2)
Supplement: Peer review history

# Reviewer report: Reviewer response for version 2

**DOI:** 10.21956/mep.22055.r38518

**Published:** 2024-08-29

**Authors:** Jennifer Hadjiev

**Affiliations:** 1 Medical College of Wisconsin, Milwaukee, USA

Great revision!

Have any limitations of the research been acknowledged?

Yes

Is the work clearly and accurately presented and does it cite the current literature?

Yes

If applicable, is the statistical analysis and its interpretation appropriate?

Yes

Are all the source data underlying the results available to ensure full reproducibility?

Yes

Is the study design appropriate and is the work technically sound?

Yes

Are the conclusions drawn adequately supported by the results?

Yes

Are sufficient details of methods and analysis provided to allow replication by others?

Yes

Reviewer Expertise:

pediatric hospital medicine, medical education

I confirm that I have read this submission and believe that I have an appropriate level of expertise to confirm that it is of an acceptable scientific standard.

# Reviewer report: Reviewer response for version 2

**DOI:** 10.21956/mep.22055.r38517

**Published:** 2024-08-23

**Authors:** Kris Rooney, Michael Schwartz

**Affiliations:** 1 Pediatrics, Lehigh Valley Health Network, Allentown, Pennsylvania, USA; 2 University of South Florida, Tampa, Florida, USA

No further comments. Appreciate the revisions.

Have any limitations of the research been acknowledged?

Partly

Is the work clearly and accurately presented and does it cite the current literature?

Yes

If applicable, is the statistical analysis and its interpretation appropriate?

Partly

Are all the source data underlying the results available to ensure full reproducibility?

No

Is the study design appropriate and is the work technically sound?

Yes

Are the conclusions drawn adequately supported by the results?

Partly

Are sufficient details of methods and analysis provided to allow replication by others?

Partly

Reviewer Expertise:

Graduate medical education

We confirm that we have read this submission and believe that we have an appropriate level of expertise to confirm that it is of an acceptable scientific standard.

# Reviewer report: Reviewer response for version 1

**DOI:** 10.21956/mep.21289.r35547

**Published:** 2024-02-09

**Authors:** Kris Rooney, Michael Schwartz

**Affiliations:** 1 Pediatrics, Lehigh Valley Health Network, Allentown, Pennsylvania, USA; 2 University of South Florida, Tampa, Florida, USA

In this study, the authors provide reasonable rationale for their primary study question, "Is USMLE step 2 scores predictive of success in pediatric residency training?". The main hypothesis is that USMLE Step 2 scores are signifcantly correlated with ACGME milestones. Secondary hypothesese include significant correlations between each of USMLE Step 1, USMLE Step 2, ITE as PGY-1, ITE as PGY-3, and ABP certifying exam. 

 This is a retrospective cohort design of 188 pediatric residents at UPMC between 2012-2017. ACGME milestones were utilized as a measure of resident "success". It is unclear on how the "milestone rating score" was obtained:

 - Was it an average across each of the 6 domains and then an average of all 21 sub-competencies?

 - Was there any correction for the 2 milestones with a range of 1-4 vs the others with a range of 1-5?

 - Were there any milestones scored as "not yet assessable"?

 We would like to see these questions answered to guide in the reproducibility of the study as well as further understanding of the meaningfulness of the final conclusions. 

 There was a well-defined cohort at a single residency program and subjects were at similar points in their training. Follow-up time, however, was not sufficiently long. There were several secular trends that occurred during the study years which led to signficant missing data:

 - Milestone reporting was only available for 129/188 end of year PGY-1 residents. 

 - Milestone reporting was only available for 122/188 end of year PGY-3 residents. 

 - ITE exam results were only available for 74/188 residents in PGY-1 year

 - ITE exam results were only available for 122/188 residents in PGY-3 year. 

 Upon review of Figure 1 and Figure 2, it is clear that Step 1, Step 2, ITE year 1, and ABP score all correlate with each other, and that milestone domains correlate with each other. The tools to measure these two types of data (exam results and milestone reporting) are vastly different. Is it fair to correlate a continuous variable such as is reported in exams with an ordinal variable as is reported in milestones? 

 To address the issue of missing data as described above, we would have liked to have seen a sensitivity analysis using only those residents with complete data to see how this affects correlation. 

 We also would have liked to have seen a supplementary table that includes actual correlations for each of the 21 milestone subcompetencies. 

 The number of ABP board exam results was also not reported. 

 The authors conclude a lack of correlation between USMLE Step 2 scores and ACGME milestones. Unfortunately, the limitations stated above may have mistakenly led to this conclusion. The authors do identify inherent limitations with regards to their study using the single pediatric program population over a short period of time. This results in lower generalizability not only in study subjects, but in milestone scoring methodology implemented at that single center. 

 We feel that by addressing the questions and providing the additional data requested above, our recommendation would easily shift from approved with reservations to approved.

Have any limitations of the research been acknowledged?

Partly

Is the work clearly and accurately presented and does it cite the current literature?

Yes

If applicable, is the statistical analysis and its interpretation appropriate?

Partly

Are all the source data underlying the results available to ensure full reproducibility?

No

Is the study design appropriate and is the work technically sound?

Yes

Are the conclusions drawn adequately supported by the results?

Partly

Are sufficient details of methods and analysis provided to allow replication by others?

Partly

Reviewer Expertise:

Graduate medical education

We confirm that we have read this submission and believe that we have an appropriate level of expertise to confirm that it is of an acceptable scientific standard, however we have significant reservations, as outlined above.

# Response

**Published:** 2024-08-13

**Authors:** Benjamin Miller

**Affiliations:** Pediatrics - School of Medicine, University of Pittsburgh, Pittsburgh, Pennsylvania, USA

**Reviewer’s comment:** 

**It is unclear on how the "milestone rating score" was obtained:**

- **Reviewer’s comment:** **Was it an average across each of the 6 domains and then an average of all 21 sub-competencies?**

**Response: **Yes.​

- **Reviewer’s comment:** **Was there any correction for the 2 milestones with a range of 1-4 vs the others with a range of 1-5?**

**Response: **No. Those 3 competencies (PC4, SBP1, and SBP3) were averaged in with the others with a maximum value of 4. As this is a comparison across residents using the same scale, this would not produce any differences in correlation coefficients.

- **Reviewer’s comment:** **Were there any milestones scored as "not yet assessable"?**

**Response:**No.​​​​​

- **Reviewer’s comment:** **We would like to see these questions answered to guide in the reproducibility of the study as well as further understanding of the meaningfulness of the final conclusions. **

**Reviewer’s comment:** **There was a well-defined cohort at a single residency program and subjects were at similar points in their training. Follow-up time, however, was not sufficiently long. There were several secular trends that occurred during the study years which led to significant missing data:**

- **Milestone reporting was only available for 129/188 end of year PGY-1 residents. **
- **Milestone reporting was only available for 122/188 end of year PGY-3 residents. **
- **ITE exam results were only available for 74/188 residents in PGY-1 year**
- **ITE exam results were only available for 122/188 residents in PGY-3 year. **

**Upon review of Figure 1 and Figure 2, it is clear that Step 1, Step 2, ITE year 1, and ABP score all correlate with each other, and that milestone domains correlate with each other. The tools to measure these two types of data (exam results and milestone reporting) are vastly different. Is it fair to correlate a continuous variable such as is reported in exams with an ordinal variable as is reported in milestones?**

**Response: **We believe this approach is fair. Yes, the tools are vastly different, which is addressed in the manuscript (as well as the point that different institutions arrive at milestones ratings differently). Concluding that these methods as surrogates for clinical competence (or the potential thereof) measure different things is the crux of the manuscript. Regarding the question on continuous versus ordinal variables, numerous other publications evaluating milestones data have treated them like continuous variables. Additionally, Stat Pearls from NCBI states: “Likert items can serve as ordinal variables, but the Likert scale, the result of adding all the items, can be treated as a continuous variable.” ( Types of Variables and Commonly Used Statistical Designs - StatPearls - NCBI Bookshelf (nih.gov))

**Reviewer’s comment: To address the issue of missing data as described above, we would have liked to have seen a sensitivity analysis using only those residents with complete data to see how this affects correlation.**

**Response: **These data are limited by non-random missingness, as described in the manuscript.  A sensitivity analysis was performed and demonstrated that the correlations were consistent when individuals with missing data were excluded.

**Reviewer’s comment:** **We also would have liked to have seen a supplementary table that includes actual correlations for each of the 21 milestone subcompetencies.**

**Response: **These supplemental figures can be found at 10.6084/m9.figshare.26598628

**Reviewer’s comment:** **The number of ABP board exam results was also not reported.**

**Response: **ABP scores were available for 101/188 residents. This has been added to the manuscript.

**Reviewer’s comment:** **The authors conclude a lack of correlation between USMLE Step 2 scores and ACGME milestones. Unfortunately, the limitations stated above may have mistakenly led to this conclusion. The authors do identify inherent limitations with regards to their study using the single pediatric program population over a short period of time. This results in lower generalizability not only in study subjects, but in milestone scoring methodology implemented at that single center.**

**We feel that by addressing the questions and providing the additional data requested above, our recommendation would easily shift from approved with reservations to approved.**

**Response: **Thank you for these thoughtful comments and suggestions. We hope you find these answers and revisions appropriately satisfactory.

# Reviewer report: Reviewer response for version 1

**DOI:** 10.21956/mep.21289.r35548

**Published:** 2023-12-21

**Authors:** Jennifer Hadjiev

**Affiliations:** 1 Medical College of Wisconsin, Milwaukee, USA

This is a very insightful and well-written retrospective cohort study examining the correlation, or lack there of, between pediatric residency applicants’ quantitative scores on the United States Medical Licensing Exam Step 2 Clinical Knowledge examination and their subsequent performance in residency training based on the Accreditation Council for Graduate Medical Education Milestones.  Although prior studies have looked at the concerns about the limitations of USMLE scores as predictors of success in residency training, this study seems very novel in specifically examining the correlation between Step 2 CK and Pediatric residency milestones.

 The study design seems very appropriate with  a good sample size and consistency in obtaining the different variables such as standardized test scores, ITE, and milestone ratings.  The details of the methods and analysis were clear and the statistical analysis and its interpretation seems appropriate. The heatmaps (Figures 1 and 2) were a nice addition and a visually appealing way to understand the results. 

 I appreciate that the major limitations of the study were addressed.  Expanding this single center study to more institutions would be interesting, and I would like to see how/if the results from pediatric residencies differ from the results in other subspecialties as well.  I also think the subjectivity in determining individual pediatric milestones is also a major limitation here and was addressed.  As stated in the article, the authors tried to minimize confounding factors and mitigate potential bias, but the collection process has not been standardized among programs.  But other than program directors and associate program directors, scorers generally don’t have access to residents’ standardized test scores, so there should be little bias from that standpoint.  However, milestone ratings from various individuals may range significantly, and that fact was not studied/commented on in the study.  It was only stated that “milestones data are generated directly from evaluations by attending physicians, pediatric fellows, supervising residents, medical students, and nurses…These data are then combined to create an overall average Milestone rating across all competencies, as well as an average for each domain of competence.”  Perhaps a future step or suggestion for other studies is to look at inter-rater reliability of various scorers of the individual milestones.

 Overall, I fully support this article with very minimal (if any) revisions.  Thank you for this insightful study and for inviting me to be a reviewer.

Have any limitations of the research been acknowledged?

Yes

Is the work clearly and accurately presented and does it cite the current literature?

Yes

If applicable, is the statistical analysis and its interpretation appropriate?

Yes

Are all the source data underlying the results available to ensure full reproducibility?

Yes

Is the study design appropriate and is the work technically sound?

Yes

Are the conclusions drawn adequately supported by the results?

Yes

Are sufficient details of methods and analysis provided to allow replication by others?

Yes

Reviewer Expertise:

pediatric hospital medicine, medical education

I confirm that I have read this submission and believe that I have an appropriate level of expertise to confirm that it is of an acceptable scientific standard.
